# A statistical approach to enhance the productivity of Streptomyces baarensis MH-133 for bioactive compounds

**DOI:** 10.1016/j.synbio.2024.01.012

**Published:** 2024-02-08

**Authors:** Mohamed H. Kalaba, Gamal M. El-Sherbiny, Osama M. Darwesh, Saad A. Moghannem

**Affiliations:** a Botany and Microbiology Department, Faculty of Science (Boys), Al-Azhar University, Cairo 11884, Egypt; b Agricultural Microbiology Department, National Research Centre, Dokki, Cairo, Egypt

**Keywords:** *S. baarensis* MH-133, Placket-Burman design, Box-Behnken design, ESKAPE, Antibacterial activity

## Abstract

The goal of this study was to use statistical optimization to change the nutritional and environmental conditions so that *Streptomyces baarensis* MH-133 could make more active metabolites. Twelve trials were used to screen for critical variables influencing productivity using the Placket-Burman Design method. *S. baarensis* MH-133 is significantly influenced by elicitation, yeast extract, inoculum size, and incubation period in terms of antibacterial activity. A total of 27 experimental trials with various combinations of these factors were used to carry out the response surface technique using the Box-Behnken design. The analyses revealed that the model was highly significant (p < 0.001), with a lack-of-fit of 0.212 and a coefficient determination (R2) of 0.9224. Additionally, the model predicted that the response as inhibition zone diameter would reach a value of 27 mm. Under optimal conditions, *S. baarensis* MH-133 produced 18.0 g of crude extract to each 35L and was purified with column chromatography. The active fraction exhibiting antibacterial activity was characterized using spectroscopic analysis. The MIC and MBC values varied between 37.5 and 300 μg/ml and 75 and 300 μg/ml, respectively. In conclusion, the biostatistical optimization of the active fraction critical variables, including environmental and nutritional conditions, enhances the production of bioactive molecules by *Streptomyces* species.

## 1 Introduction

ESKAPE pathogens, a class of bacteria, are multidrug-resistant and present a significant hazard to human health. *Enterococcus faecalis, Staphylococcus aureus, Klebsiella pneumoniae, Acinetobacter baumannii, Pseudomonas aeruginosa*, and *Enterobacter* species comprise the acronym ESKAPE. The aforementioned bacteria have been linked to the most severe potential for antibiotic resistance to affect clinical and economic systems [1,2]. ESKAPE pathogens are included on the list of antibiotic-resistant “priority pathogens” published by the World Health Organization [3]. High mortality rates and severe infections are directly attributable to the fact that the majority of these microorganisms are capable of surviving in the hospital environment via biofilm formation or the capacity to withstand stress conditions (e.g., the presence of disinfectants). The prevalence of healthcare-associated coinfections has been significantly influenced by the establishment of these pathogens in hospitals in recent times, amidst the severe acute respiratory syndrome coronavirus 2 (SARS-CoV-2) pandemic [4]**.** Additionally, antibiotic resistance has a huge economic effect, with estimates indicating that it might cost the world economy up to $100 trillion by 2050 [5]. To lessen the economic cost of antibiotic resistance, new antibiotics are required. This necessitates the development of novel antibiotics immediately [6].

Researchers have implemented multiple emerging strategies to combat multidrug-resistant (MDR) ESKAPE pathogens. Researchers have investigated antibiotic combinations and adjuvants, bacteriophages, antimicrobial peptides, nanoparticles, and plant extracts as potential treatments for ESKAPE infections [7]. Actinomycetes, specifically *Streptomyces*, are a category of bacteria that have been linked to the production of many antibiotics used today [8]. The obtaining of innovative antibiotics from these bacteria, however, has slowed in recent years. This condition may be the outcome of antibiotic discovery efforts, usually focusing on screening culturable environmental microorganisms for antimicrobial chemicals, such as bacteria from soil. This virtually always results in the re-isolation of previously isolated microorganisms [9]. To solve these difficulties, we search for new isolates in locations that are often unreachable by human activity. In addition, antibiotic-resistant strains have also been employed as test strains. These procedures are particularly efficient in excluding previously known compounds or chemicals to which bacteria have evolved resistance. After following these steps, we were able to obtain a type of *Streptomyces* that can kill the ESKAPE pathogen group [10]. *Streptomyces* are important in the pharmaceutical and biotechnology industries, making their productivity optimization a key focus for researchers [11].

*Streptomyces* species have been regarded as repositories of an extensive variety of natural products due to the complexity and potency of their secondary metabolism. Streptomyces is responsible for the production of an estimated 100,000 antibiotic compounds, which account for 70–80% of all naturally occurring bioactive products with pharmacological or agrochemical applications [12,13]. A diverse array of natural products, such as terpenes, macrolides, tetracyclines, aminoglycosides, glycopeptides, ansamycins, and aminoglycosides, are produced by *Streptomyces* [14]. *Streptomyces*-derived bioactive natural products exhibit a diverse array of potential uses, such as cytotoxic, antimicrobial, antiviral, antitumor, antihypertensive, immunosuppressive, insecticidal, antioxidative, plant growth-promoting, and herbicidal properties [15].

A significant factor impeding the ability of microorganisms, especially *Streptomyces,* to synthesize numerous metabolic substances is the inadequate nutritional and environmental conditions that do not meet their requirements [16]. *Streptomyces* productivity optimization is primarily concerned with maximizing the yield of bioactive compounds such as antibiotics. Statistical optimization designs make it possible to increase the yield of target compounds by providing a methodical approach that takes into account all pertinent variables. The existence of certain statistical models, including the Box-Behnken Design (BBD) and Plackett-Burman Design (PBD), significantly aids in this regard [17]. PBD is a useful tool for identifying and filtering critical factors that affect microbial productivity. It enables the concurrent examination of numerous variables while minimizing the number of experiments required. When researchers figure out which variables are most important, like agitation speed, pH, nutrient composition, and temperature, they can quickly focus on the most important ones to get even better results [18]. Once the important components have been determined, an approach known as BBD may be used to optimize and adjust the values of these factors. The use of BBD is especially advantageous in the context of systems that include numerous variables and exhibit intermediate response surfaces. This methodology facilitates the identification of optimum factor designs that may substantially improve the productivity of *Streptomyces*, while simultaneously reducing the need for many experimental trials [19]. Herein, the goal of this work is to use statistical optimization methods to adjust the nutritional and environmental parameters required to maximize the production of *S. baarnensis* *MH-133* metabolites with antibacterial activity. The study also intends to characterize this fractionated metabolite and determine its minimum inhibitory concentration (MIC), which is the minimum concentration at which bacterial growth is inhibited, and minimum bactericidal concentration (MBC) is defined as the smallest concentration at which the inoculated bacteria is completely killed.

## 2 Materials and methods

We have already found and tested our *S. baarnensis* MH-133 for its ability to kill MDR-ESKAPE pathogenic bacteria, such as *Enterococcus faecalis*, methicillin-resistant *Staphylococcus aureus* (MRSA), *Klebsiella pneumonia, Acinetobacter baumanii, Pseudomonus aeruginosa, E. coli*, and *Enterobacter cloacae*. It has also been tested against the standard strains *Bacillus subtilis* ATCC-6633 and *Salmonella typhi* ATCC-6539. Furthermore, one factor at a time (OFAT) optimization and biotic elicitation were used to boost the productivity of *S. baarnensis* MH-133 in previous studies [10,20].

### 2.1 Optimization of process parameters

The optimization procedure using a statistical technique was conducted in a two-step manner. The first phase was the identification of the constituents within the medium that have a substantial influence on the synthesis of bioactive metabolites using the PBD. The subsequent procedure included the identification of the interaction and optimal levels via the use of BBD.

### 2.2 Screening for crucial variables using PBD

Plackett-Burman design (PBD) was utilized to identify the most influential environmental and nutritional factors that could affect the antibacterial action of *S. baarnensis* MH-133. Minitab 18 was used for both the setup of the experiments and the examination of the data from the experiments. Eleven factors (independent variables) that could potentially influence *S. baarnensis* MH-133 production of antibacterial metabolites were evaluated in this experimental design using two levels of representation: low and high, in twelve designated trials. Table 1**.** The variables comprised pH, incubation period, cultivation method, inoculum size, incubation temperature, magnesium chloride, casein, biotic elicitation, starch, yeast extract, and sodium chloride. The experiments were replicated twice, and the response was measured as the mean of inhibition zone diameter, indicating antibacterial activity against *K. pneumonia*. The experimental design of Plackett-Burman is founded upon the first-order as a model of ESKAPE pathogens:

Y = β0 +∑ βi xi,

**Table 1:** Plackett-Burman design matrix to screen beneficial parameters for *S. baarnensis* MH-133 antibacterial metabolite synthesis.

Run no.	pH	Incubation time (day)	Cultivation method (rpm)	Inoculum size (V/V)	Temperature (°C)	Magnesium chloride (g/l)	Casein (g/l)	Yeast extract (g/l)	Starch (g/l)	Elicitation (V/V)	NaCl (g/l)	inhibition zone diameter (mm)
1	9	12	0	4	25	13.2	0.5	3	15	6	15	16.5
2	7	12	200	8	25	4.4	0.5	3	5	10	15	9.5
3	7	12	200	4	35	13.2	1.5	1	5	6	15	13
4	9	4	200	8	25	13.2	1.5	3	5	6	5	16.5
5	9	12	200	4	25	4.4	1.5	1	15	10	5	0
6	7	4	0	8	25	13.2	1.5	1	15	10	15	0
7	7	4	200	4	35	13.2	0.5	3	15	10	5	0
8	7	4	0	4	25	4.4	0.5	1	5	6	5	9.5
9	9	4	200	8	35	4.4	0.5	1	15	6	15	13.5
10	9	12	0	8	35	13.2	0.5	1	5	10	5	0
11	9	4	0	4	35	4.4	1.5	3	5	10	15	0
12	7	12	0	8	35	4.4	1.5	3	15	6	5	21

The response variable Y represents antibacterial activity, the model's intercept is β0, the linear coefficient is βi, and the independent variable's level is xi [21]. The Plackett-Burman design was subjected to regression and Analysis of variance (ANOVA) analyses. The Box-Behnken design was utilized to optimize the variables that were determined to have a significant impact on antibacterial activity (p < 0.05) based on the regression analysis.

### 2.3 Response surface methodology (RSM)

After determining the parameters influencing antibacterial action, the BBD was utilized to optimize the levels of significant variables such as inoculum size, incubation time, elicitation, and yeast extract in 27 runs. Table 2. The experimental strategy for this research included 27 runs, and the independent variables were evaluated at three distinct levels: low, high, and center points. The experimental results of RSM were fitted using the response surface regression method and the subsequent second-order polynomial equation:

Y = β_0_ + Σ β_i_X_i_ + Σ β_ii_X_i_^2^ + Σ β_ij_X_i_X_j_,

**Table 2:** BBD for optimization of the factors affecting the production of antibacterial metabolites by *S. baarnensis* MH-133.

Run Order	Incubation time (day)	Inoculum size % (v/v)	Elicitation % (v/v)	yeast extract (g/l)	Inhibition zone diameter (mm)
1	4	8	8	2	13
2	12	8	8	2	21
3	4	6	6	2	12
4	12	6	8	1	14
5	4	6	8	1	10
6	8	6	8	2	16
7	8	6	8	2	15
8	4	4	8	2	11
9	8	6	6	1	13
10	12	4	8	2	17
11	8	4	8	3	14
12	8	4	8	1	12
13	12	6	10	2	17
14	8	8	8	3	18
15	8	8	8	1	13
16	8	6	10	1	14
17	12	6	6	2	21
18	4	6	8	3	13
19	4	6	10	2	11
20	8	4	6	2	16
21	8	8	6	2	20
22	8	6	6	3	23
23	8	4	10	2	10
24	8	6	8	2	16
25	8	6	10	3	12
26	8	8	10	2	14
27	12	6	8	3	21

The response variable Y represents the inhibition zone diameter (mm), the interception coefficient is denoted as β0, the coefficients of the linear effect and quadratic effect are denoted as βi and βii, respectively, and the cross-product coefficients are denoted as β ij. The independent variables XIXj have an impact on the response variable Y. An analysis of variance (ANOVA) was conducted to evaluate the statistical adequacy of the model. The Fisher's test and its associated probability were employed to validate the significance of the overall model. Utilizing the coefficient of determination (R2) and adjusted R2, the accuracy of the polynomial model equation was evaluated [22].

### 2.4 Validation of optimization

The statistical model of optimization was experimentally confirmed by cultivating *S. baarnensis* MH-133 on an adapted marine broth medium (MB) for 12 days while taking into consideration the optimal levels of variables derived from the Box-Behnken design. After incubation, antibacterial activity against *K. pneumonia* was determined using the agar diffusion technique [23]. Furthermore, the antibacterial activity of the unoptimized, optimized culture using OFAT, and optimized cultures using statistical methods was compared.

### 2.5 Production and extraction of bioactive metabolite(s) produced by *S. baarnensis* MH-133

The optimized MB medium was prepared, inoculated with the seed culture of *S. baarnensis* MH-133, and incubated under optimized environmental conditions. Following incubation, the broth was filtered via a cotton layer to eliminate spores and mycelium before being centrifuged at 5000 rpm for 20 min to separate planktonic cells. To extract bioactive metabolites, the clear filtrate was adjusted to pH 7.0, and equal volumes of the culture filtrate and various solvents (*n*-hexane, cyclohexane, petroleum ether, benzene, toluene, diethyl ether, chloroform, ethyl acetate, acetone, methanol, ethanol, isopropanol, and n-butanol) were chosen. For 20 min, each solvent and clear filtrate were forcefully combined. After shaking, the mix was allowed to settle in a separation funnel and form both a distinct aqueous and organic layer. The organic layer was collected and concentrated by rotational evaporation of the solvent (Heidolph - laborota 4000 rotovap, USA) until deep red residues were produced. By using the agar disk diffusion technique, the crude extracts of each solvent were examined for their efficacy against *K. pneumonia* [24,25].

### 2.6 Selection of the appropriate solvent system for the separation of a crude extract by thin-layer chromatography (TLC)

By utilizing TLC, the optimal solvent for achieving a successful separation was determined. The crude extract was dissolved in ethyl acetate and applied onto thin-layer chromatography (TLC) plates (TLC 20 × 3 cm, silica gel 60F 254, Merck Co, USA) using various mobile phase solvents: Methanol: Chloroform (1:1), ethyl acetate: methanol (1:1), and diethyl ether: ethanol (1:1). This was done to identify the optimal solvent system for separating the bioactive compounds and to assess their solubility with the crude extract. After the operating process had been concluded, the plates underwent a drying procedure. After that, ultraviolet (UV) light was used to look at the chromatograms on the thin-layer chromatography (TLC) plates and figure out where the spots were [26].

### 2.7 Purification of crude extract using the column-chromatography technique

The crude extract was purified through the utilization of column chromatography with silica gel of column chromatography grade (60–120 mesh, Mumbai). The 2.5 × 50 cm column underwent a rinsing process using acetone. Following the dehydrating process, silica gel was inserted into the column. As a solvent system, ethyl acetate: methanol in various proportions (9:1, 8:2, 7:3, 6:4, 5:5, and 4:6) was utilized. The packed column was loaded with 5 mL of dissolved crude extract (2.5 gm/5 ml of eluting solvent) and eluted with the solvent system. To capture the eluted fractions, dry-clean glass containers were positioned at the base of the column. The antibacterial action of these fractions was estimated by employing the paper disk method. The fractions that demonstrated efficacy against *K. pneumonia* were subsequently subjected to purity analysis using a TLC plate, and the retention factor (Rf) was also determined [27].

### 2.8 Determination of (MIC) and (MBC) of purified compound (Ka) using the microdilution method

The antibacterial action of purified compound (Ka) was assessed based on CLSI, [28]. In brief, two-fold dilution series were prepared to achieve a decreasing concentration ranging from 1200 to 37.5 μg/ml) of purified compound (Ka), which was dissolved in dimethyl sulfoxide (DMSO) (2400 μg/ml stock solution). In a 96-well microtiter plate, 150 μl of double-strength Muller Hinton broth medium were put in each plate well, then 150 μl of Ka was loaded in each well of the first raw of the plate and mixed well to make the final concentration equal to 1200 μg/ml. Then, 150 μl of this well was transferred to the well of the next raw to make the final concentration equal to 600 μg/ml, and so on until reaching the final concentration, which equals 37.5 μg/ml. For the bacterial strains under investigation, broth cultures were set up overnight. An inoculum containing 5% (V/V) (OD = 0.5 McFarland standard) of each strain was introduced into its corresponding well. Streptomycin was included in the assays as an antibiotic-positive control at the same concentrations. One well at each antibiotic concentration was inoculated with medium alone as a sterility control. To establish a growth control, wells devoid of any of the tested compounds were inoculated with identical inoculum sizes for each test strain. A negative control solution was prepared by serial dilution of dimethyl sulfoxide (DMSO) solution (two-fold dilution series) and inoculated with the test strains. This approach eliminated the influence of the DMSO in which Ka was dissolved. Following a 24-h statically incubated at 37 °C, each plate was read at 610 nm using an ELISA reader [29]. The MIC is operationally defined as the concentration at which bacterial growth is not visually seen in comparison to positive control. Following the determination of the MIC values of the tested metabolite, a volume of 500 μl of the tested broth was applied onto sterile Mueller Hinton Agar (MHA) plates. These plates were then incubated at a temperature of 37 °C for 24 h. The purpose of this incubation was to assess the bactericidal impact of Ka and streptomycin on each strain.

#### 2.8.1 Identification of the purified compound (Ka) obtained from *S. baarnensis* MH-133

The active purified fraction was characterized according to Janardhan et al. [30], utilizing spectroscopic analysis such as ultraviolet (UV (160A-Shimadzu), Infrared IR (Matson Satellite 113 spectrometer) at the National Research Center, Giza. Proton nuclear magnetic resonance (1HNMR) (various Mercury −300BB/MHz NMR spectrometer) at the faculty of pharmacy, Ainshams University, Cairo, in addition to Mass Spectrum (Direct Inlet part DI-50 to mass analyzer in Shimadzu GC-MS-QP5050 Thermo Scientific Prop) at Regional Center of Mycology and Biotechnology.

## 3 Results

### 3.1 Screening for essential factors affecting the productivity of *S. baarnensis* MH-133 using Placket-Burman Design (PBD)

**Fig. 1:**
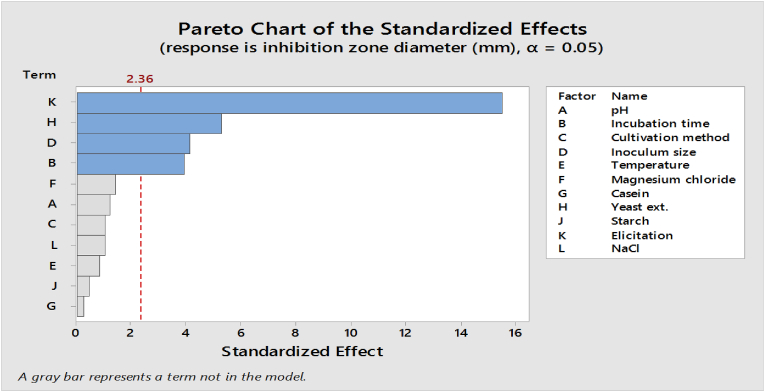
Pareto plot of Placket–Burman design shows eleven factors that have positive and negative effects on antibacterial activity tested on *K. pneumonia.* Based on a threshold value, the red line in a Pareto plot for a Plackett-Burman design is determined. The threshold value is commonly established at the point where the most influential factors can be economically detected, under the assumption that all interactions are insignificant in comparison to the limited number of significant main effects. Significant factors are those whose effects surpass the threshold denoted by the red line; those whose effects fall short of the line are regarded as non-significant. Therefore, in the Pareto diagram, the red line functions as a visual assistance to distinguish the influencing factors from the non-influencing ones.

The PBD design has shown its efficacy as a beneficial tool for screening media components and growth conditions in many bioprocesses, including antibiotic synthesis. In the optimization process, a total of 11 factors were chosen, each with two distinct levels. A series of 12 experiments were conducted to ascertain the medium ingredients and conditions that have a substantial effect on the antibacterial activity shown by *S. baarnensis* MH-133. The response of these trials was tested against *K. pneumonia*. The maximum antibacterial activity against *K. pneumonia* was obtained from trial number (12), where the inhibition zone diameter was 21 mm (Table 1). The data indicate that elicitation, yeast extract, inoculum size, and incubation time have a significant effect on antibacterial activity by *S. baarnensis* MH-133 in comparison with other factors. The affecting variables were further confirmed by the Pareto chart, and the factors that had an impact were verified via the use of a Pareto chart (Fig. 1). This chart displayed the absolute values of the impacts and included a standardized reference line based on a 95% confidence level. The variables that exhibited values beyond the reference line were deemed to be statistically significant (p < 0.05). Furthermore, the impact of the characteristics, whether they exhibit synergistic or antagonistic behavior, was comprehended by using the coefficients or effects. The presence of a positive regression coefficient in the tabular columns signifies a synergistic impact on the antibacterial activity, whereas a negative coefficient indicates an antagonistic effect, as shown in the provided list (Table 3).

**Table 3:** Analysis of variance of a screening model for effecting variables on antibacterial activity tested against *K. pneumonia* using PBD.

Source	DF *	Seq SS	Contribution	Adj SS	Adj MS	F-Value	P-Value
Model	4	676.58	97.74%	676.58	169.146	75.68	0
Linear	4	676.58	97.74%	676.58	169.146	75.68	0
Incubation time	1	35.02	5.06%	35.02	35.021	15.67	0.005
Inoculum size	1	38.52	5.56%	38.52	38.521	17.23	0.004
Yeast ext.	1	63.02	9.10%	63.02	63.021	28.2	0.001
Elicitation	1	540.02	78.01%	540.02	540.021	241.61	0
Error	7	15.65	2.26%	15.65	2.235		
Total	11	692.23	100.00%				

Coded Coefficients						
Term	Coef	SE Coef	95% CI	T-Value	P-Value	VIF
Constant	8.292	0.432	(7.271, 9.312)	19.21	0	
Incubation time	1.708	0.432	(0.688, 2.729)	3.96	0.005	1
Inoculum size	1.792	0.432	(0.771, 2.812)	4.15	0.004	1
Yeast ext.	2.292	0.432	(1.271, 3.312)	5.31	0.001	1
Elicitation	−6.708	0.432	(-7.729, −5.688)	−15.54	0	1

^a^DF: Degrees of Freedom, Seq SS: Sequential Sum of Squares, Contribution: Contribution to the model, Adj SS: Adjusted Sum of Squares, Adj MS: Adjusted Mean Square, F-Value: F-Statistic, P-Value: Probability Value, Coef: Coefficient estimate, SE Coef: Standard error of the coefficient estimate, 95% CI: 95% confidence interval for the coefficient estimate, T-Value: The T-value for the hypothesis test of the coefficient being 0, VIF: Variance Inflation Factor.

### 3.2 Response surface methodology

**Fig. 2:**
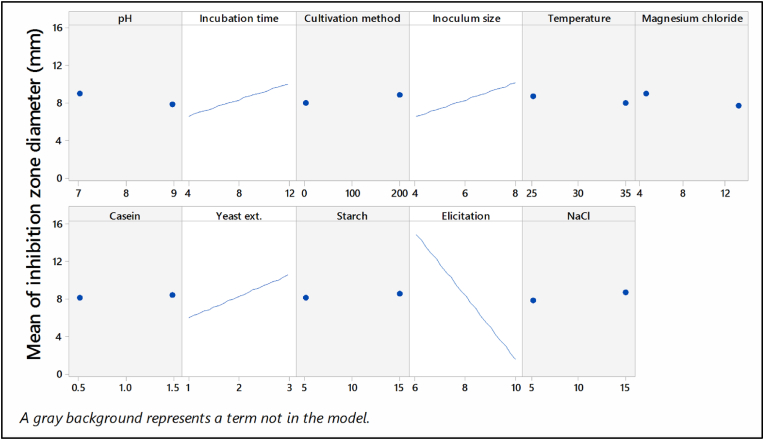
Main effect plot for inhibition zone diameter (mm).

Based on the confidence level, the most significant variables-elicitation, inoculum size, incubation time, and yeast extract-were chosen as influencing factors for further optimization by a RSM with BBD. In this study, a total of 27 experimental trials with different combinations of these factors were performed, where the remaining components were held constant at the indicated level as shown in Fig. 2, and their effect on antibacterial activity was determined. The maximum antibacterial activity was observed in trial no. 22 with an inhibition zone diameter of 23 mm against *K. pneumonia* Table 2. The actual response (antibacterial activity) was analyzed by Minitab 18 software. The coefficients of the quadratic regression equation were computed, and the data was then fitted to a polynomial equation of second order:

ActivityagainstK.pneumonia=‐15.68+0.8542Incubationtime(day)+0.792Inoculumsize%(v/v)+1.875Elicitation%(v/v)+14.08yeastextract(g/l)‐1.500Elicitation%(v/v)*yeastextract(g/l)

**Fig. 3:**
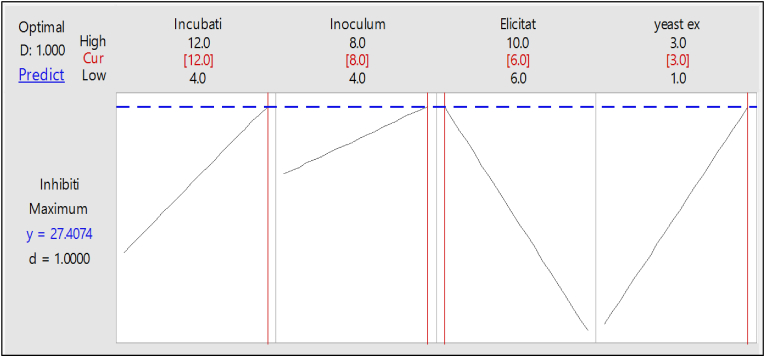
Response optimizer showing the ideal level of factors affecting antibacterial action by *S. baarnensis* MH-133 against *K. pneumonia.*

The statistical significance of Equation (1) was confirmed by the F-test, and the ANOVA for the response surface quadratic model is brief in Table 4. The model was found to be highly significant (p < 0.001), and its coefficient determination (R2) was displayed as 0.9224. This means that when the response was tested against *K. pneumonia*, 92.24% of the variability in the response could be explained by the model, and less than 8% of the total variations were not explained. The model's relevance was suggested by the adjusted determination coefficient value (Adj. R2 = 0.9039), which was in acceptable agreement with the expected R2 of 0.8749. By solving the inverse matrix using Minitab 18, the maximum antibacterial activity (inhibition zone diameter) was 27 mm as a predicted value if the filtrate of *S. baarnensis* MH-133 was tested against *K. pneumonia* Fig. 3**.**

**Table 4:** Analysis of variance and coded coefficients for the experimental results of the Box-Behnken design.

Source	DF	Adj SS	Adj MS	F-Value	P-Value
Model	5	319	63.8	49.9	0
Linear	4	283	70.75	55.33	0
Incubation time (day)	1	140.083	140.083	109.55	0
Inoculum size % (v/v)	1	30.083	30.083	23.53	0
Elicitation % (v/v)	1	60.75	60.75	47.51	0
yeast extract (g/l)	1	52.083	52.083	40.73	0
2-Way Interaction	1	36	36	28.15	0
Elicitation % (v/v)*yeast extract (g/l)	1	36	36	28.15	0
Error	21	26.852	1.279		
Lack-of-Fit	19	26.185	1.378	4.13	0.212
Pure Error	2	0.667	0.333		
Total	26	345.852			

Coded Coefficients					
Term	Coef	SE Coef	T-Value	P-Value	VIF
Constant	15.074	0.218	69.27	0	
Incubation time (day)	3.417	0.326	10.47	0	1
Inoculum size % (v/v)	1.583	0.326	4.85	0	1
Elicitation % (v/v)	−2.25	0.326	−6.89	0	1
yeast extract (g/l)	2.083	0.326	6.38	0	1
Elicitation % (v/v)*yeast extract (g/l)	−3	0.565	−5.31	0	1

### 3.3 Validation of the optimization design

The model validation was done by applying the statistically optimized conditions determined by the model, and the response was estimated in the form of inhibition zone diameter, indicating the antibacterial action of *S. baarnensis* MH-133 as shown in Table 5**.** When the filtrate of *S. baarnensis* MH-133 was tested against *K. pneumonia,* the best correlation between predicted (27 mm) and measured values validated the response model and the existence of an optimal point for antibacterial action, as evidenced by the 26 mm maximum antibacterial action obtained. From the overall assessment, 8% inoculum size, 6% elicitation, yeast extract of 3 g/l in modified marine broth medium, and incubation for 12 days may be regarded as the adjusted conditions for increasing the antibacterial action of *S. baarnensis* MH-133. The chemical composition of the optimized modified marine broth medium and environmental conditions that may affect the productivity of *S. baarnensis* MH-133 are listed in Table 6.

**Table 5:** Comparison between antibacterial action of *S. baarnensis* MH-133 related to the culture conditions.

Culture condition	Inhibition zone diameter (mm) against *K. pneumonia*			
	Unoptimized	Optimized with OFAT	Optimized with BBD (Experimental)	Optimized with BBD (Predicted)
	14	20	26	27

(OFAT) = one factor at a time, (BBD) = Box-Behnken Design.

**Table 6:** Nutritional and environmental requirements of *S. baarnensis* MH-133 according to statistical optimization designs.

No.	Nutritional composition	(g/l)
1	Starch	5
2	Casein	1
3	Yeast Extract	3
4	Ferric Citrate	0.1
5	Sodium chloride	5
6	Magnesium Chloride	4.4
7	Sodium Sulfate	3.24
8	Calcium Chloride	1.8
9	Potassium Chloride	0.55
10	Sodium Bicarbonate	0.16
11	Potassium Bromide	0.08
12	Strontium Chloride	0.034
13	Boric Acid	0.022
14	Sodium Silicate	0.004
15	Sodium Fluoride	0.0024
16	Ammonium Nitrate	0.0016
17	Disodium Phosphate	0.008
Environmental conditions		
1	Elicitation (%v/v)	6
2	Inoculum size (%v/v)	8
3	pH	7
4	Temperature °C	25
5	Shaking (rpm)	0 (static)
6	Incubation time (day)	12

### 3.4 Production and extraction of bioactive metabolite(s) produced by *S. baarnensis* MH-133

*Streptomyces barnensis* MH-133 was subjected to submerged culture fermentation in Erlenmeyer flasks at optimized conditions. The bioactive metabolites were extracted using different solvents according to relative polarity on a graded scale. The extraction of bioactive metabolites from the cell-free filtrate of *S. baarnensis* MH-133 involved the utilization of thirteen distinct solvents. In terms of their antibacterial activity against *K. pneumonia*, the three organic solvents that extracted the bioactive metabolites from the culture filtrate the most effectively were ethyl acetate, chloroform, and diethyl ether, (Table 7). Ethyl acetate emerged as the most effective extraction solvent, exhibiting the greatest inhibition zone (16 mm) against *K. pneumonia*. 35 L of cell-free filtrate were extracted with ethyl acetate; the resulting organic phase was collected using a separating funnel and concentrated via rotary evaporator to yield 18 g of crude extract.

**Table 7:** Antibacterial Activity of different crude extracts obtained by different solvents.

No.	Solvent	Inhibition zone diameter (mm) against *K. pneumonia*
1	*n*-hexane	0
2	Cyclohexane	0
3	Petroleum ether	0
4	Benzene	0
5	Toluene	0
6	Diethyl ether	12
7	Chloroform	12
8	Ethyl acetate	16
9	Acetone	0
10	Methanol	0
11	Ethanol	0
12	Isopropanol	0
13	n- butanol	0

### 3.5 Choice of the appropriate solvent system for purification of crude extract by TLC

**Fig. 4:**
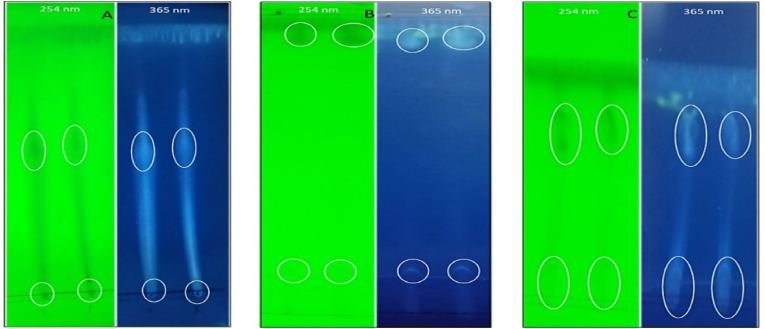
Thin Layer Chromatography (TLC) of crude extract using different solvent systems under UV light. A; methanol: chloroform, B; ethyl acetate: methanol, C; diethyl ether: ethanol.

The best solvent system used for purification should have the following criteria: solubilization of crude extract, maximum fractionation of crude extract into separate bands, and finally, the complete migration of crude extract from baseline. To choose a solvent system suitable for the purification of active compound(s), different solvent systems [methanol: chloroform (1:1), ethyl acetate: methanol (1:1), and diethyl ether: ethanol (1:1)] were used as a mobile phase, and silica gel-coated sheets were used as a stationary phase. The results indicated that the solvent system methanol: chloroform separated only one band from the crude extract, while the remaining was attached to the baseline of the TLC plate. Ethyl acetate: methanol system separated the crude extract into two bands, one band under the front line of the solvent and the other band near the base line. On the other hand; diethyl ether: ethanol system was like methanol: chloroform where it separated the crude extract into two elongated bands, one band under the front line of the solvent and the other one on the baseline. Based on the obtained findings, the solvent system, including ethyl acetate and methanol, was determined to be the most optimal choice for the purification of the active compound(s) from the crude extract by the use of column chromatography (CC) (Fig. 4).

#### 3.5.1 Purification of crude extract using column chromatography technique

**Fig. 5:**
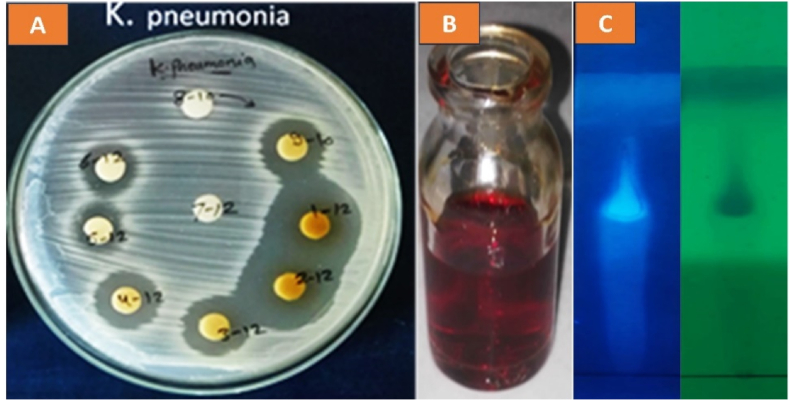
Antibacterial action of purified fractions taken by CC (A), bioactive fraction (B), TLC profile of bioactive fraction (C).

The crude extract (2.5 g) was dissolved in 5 ml of eluting solvent (ethyl acetate: methanol) and then put into a silica gel column (2.5 × 50 cm). Fifty-four fractions were collected using six gradients of the ethyl acetate: methanol solvent system. All fractions were checked for antibacterial action against *K. pneumonia*. The fractions that were eluted with ethyl acetate: methanol (4:6) (fractions 1–12 to 6–12) showed antibacterial activity, in addition to the last fraction eluted with ethyl acetate: methanol (5:5) (fractions no. 9–10), while the other fractions failed to exhibit any activity against the tested bacteria (Table 8) and (Fig. 5A)**.** The fractions obtained from column chromatography that showed antibacterial activity were tested for purity using TLC. The purified fractions showing the same pattern and purity were pooled, dried, and coded Ka for the next steps of the study. The purified fraction (Ka) was obtained in ethyl acetate: methanol (4:6) with Rf 0.6, as shown in Fig. 5B and C.

**Table 8:** Antibacterial action of different fractions taken from CC.

Solvent system	Fraction No.	Inhibition zone diameter (mm)	Solvent system	Fraction No.	Inhibition zone diameter (mm)
Ethyl acetate: methanol (9:1)	1–2	0	Ethyl acetate: methanol (6:4)	1–8	0
	2–2	0		2–8	0
	3–2	0		3–8	0
	4–2	0		4–8	0
	5–2	0		5–8	0
	6–2	0		6–8	0
	7–2	0		7–8	0
	8–2	0		8–8	0
	9–2	0		9–8	0
Ethyl acetate: methanol (8:2)	1–4	0	Ethyl acetate: methanol (5:5)	1–10	0
	2–4	0		2–10	0
	3–4	0		3–10	0
	4–4	0		4–10	0
	5–4	0		5–10	0
	6–4	0		6–10	0
	7–4	0		7–10	0
	8–4	0		8–10	0
	9–4	0		9–10	16
Ethyl acetate: methanol (7:3)	1–6	0	Ethyl acetate: methanol (4:6)	1–12	27
	2–6	0		2–12	27
	3–6	0		3–12	18
	4–6	0		4–12	16
	5–6	0		5–12	12
	6–6	0		6–12	12
	7–6	0		7–12	0
	8–6	0		8–12	0
	9–6	0		9–12	0

### 3.6 Determination of MIC and MBC of purified compound (Ka) using microdilution method

**Fig. 6:**
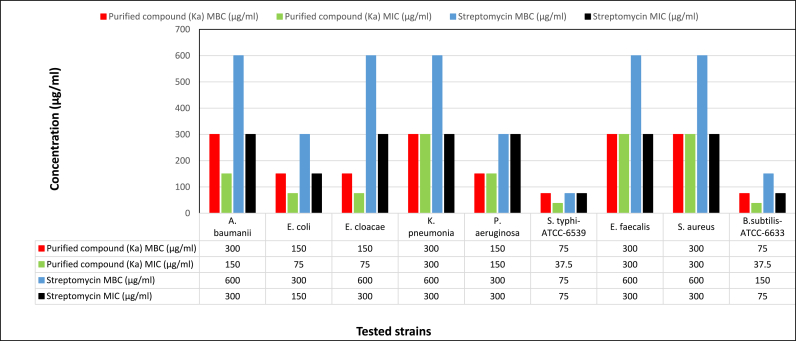
MIC and MBC of Ka and streptomycin against ESKAPE pathogenic bacteria.

In the microdilution assay, the MIC and MBC of Ka and streptomycin antibiotics were determined, and the results indicated that Ka and streptomycin both exhibit antibacterial action against the tested strains, with variable effectiveness. Fig. 6**.** The MIC values of Ka ranged from 37.5 to 300 μg/ml and the MBC values ranged from 75 to 300 μg/ml, while the MIC values of streptomycin, which was used as the control antibiotic in this assay, ranged from 75 to 300 μg/ml and the MBC values ranged from 75 to 600 μg/ml.

### 3.7 Identification of purified compound (Ka) obtained from *S. baarnensis* MH-133

**Fig. 7:**
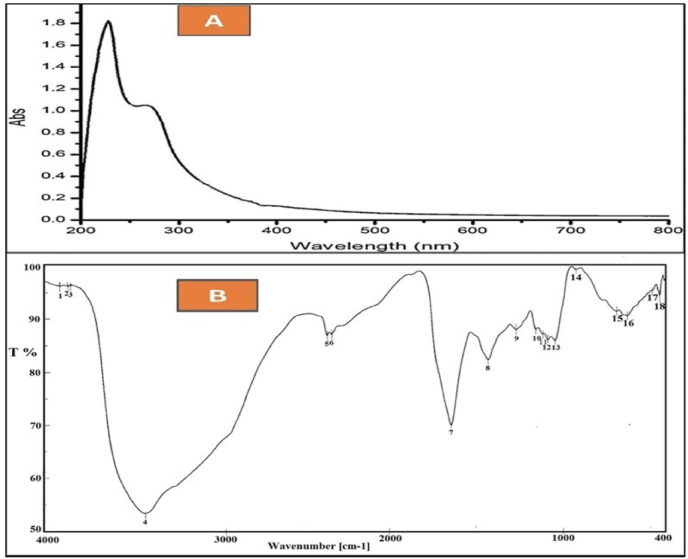
UV (MeOH) spectrum (A), FT-IR spectrum (B) of purified compound (Ka) obtained from *S. baarnensis* MH-133.

The purified compound (Ka) was obtained in the form of a reddish-brown solid, exhibiting high solubility in DMSO and methanol, moderate solubility in ethyl acetate, and no solubility in water and hexane. The Ka was characterized through different spectroscopic analyses. These analyses were ultraviolet (UV), infrared (IR), proton nuclear magnetic resonance (H NMR), and mass spectrum (MS) analysis. The compound showed a UV absorbance on TLC, which turned to blue-violet by treating it with 2 N sodium hydroxide as an indication of a peri-hydroxyquinone. The UV spectrum of Ka in Fig. 7-A displayed a characteristic peak at *λ*_max_ = 237–287 nm, referring to its aromatic nature. The dried compound was ground in KBr, and the absorbances were screened in the range of 400–4000 cm^−1^ to produce the FT-IR spectra. The IR chart of Ka demonstrates the hydroxyl group at 3421-3338 cm^−1^, the quinoid carbonyl stretch at 1637 cm^−1^, C–H_2_ stretching bending at 1420 cm^−1^, and C–O stretching peaks that are strong and typically fall between 1300 and 1000 cm^−1^, Fig. 7-B.

**Fig. 8:**
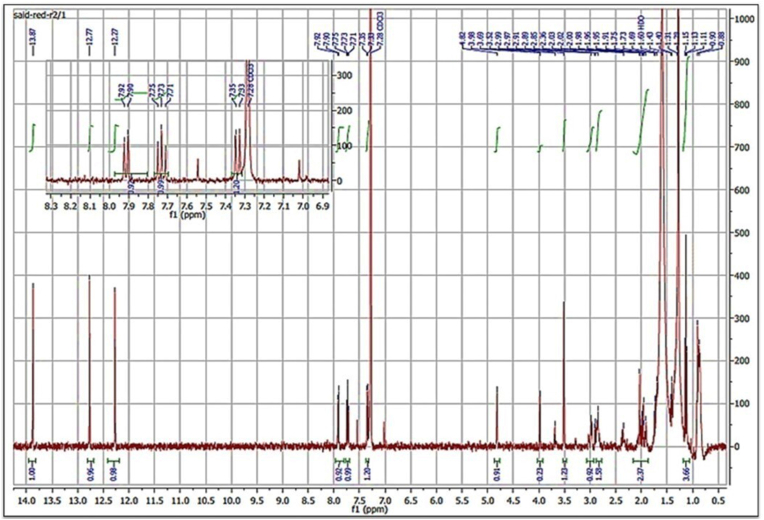
1H NMR (400 MHz, CDCl3) spectrum of Ka obtained from *S. baarnensis* MH-133.

In the downfield region, the 1H NMR spectrum of Ka manifests three singlets each of 1H at *δ* 13.87, 12.77, and 12.27 ppm, which are characteristic of peri-hydroxy groups in a quinone moiety (Fig. 8). In the aromatic region, three aromatic 1H resonances are observed at *δ* 7.91 (dd, 7.8, 1.0, H-11) ppm, and two protons act as in AB system each of 1H at *δ* 7.73 and 7.35 ppm as two ortho-coupled protons in the 1,2,3,4-tetrasubstituted phenolic ring. Moving to the right, a 1H singlet is detected at *δ* 4.82 (H-10) ppm, which can be assigned as an oxymethine group (CH(OH)). Four multiplets are displayed (each of 1H) of two vicinal methylene groups at *δ* 2.98 (Ha-7), 2.90 (Hb-7), 2.35 (Ha-8), and 2.10 (Hb-8) ppm. The first methylene group (*δ* 2.98 and 2.90) should be next to a sp2 carbon, while the other one (*δ* 2.35 and 2.10) should be next to an oxygenated quaternary sp3 carbon. In addition, a third methylene group is resonated as two multiplets between *δ* 2.00 and1.95 (H2-13) ppm and could be adjacent to a methyl group, which generates a triplet at *δ* 1.13 (H3-14) ppm to constitute an ethyl group.

**Fig. 9:**
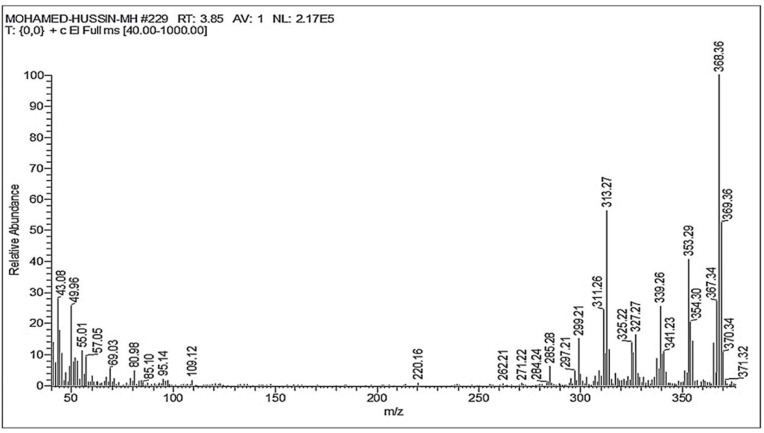
Electrospray ionization mass spectrometry of Ka obtained from *S. baarnensis* MH-133.

The molecular weight of Ka (Fig. 9) was determined to be 370 Da based on its mass spectrum. The spectrum showed a molecular ion peak at *m*/*z* (%) 370 ([M]+, 10) and a base peak at 368 ([M − 2H]+, 100).

**Fig. 10:**
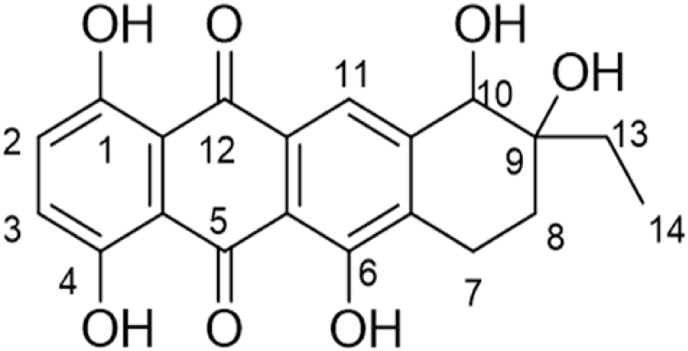
9-Ethyl-1,4,6,9,10-pentahydroxy-7,8,9,10-tetrahydrotetracene-5,12-dione (Ka).

Based on the above chromatographic and spectroscopic data of the purified compound Ka, one possible structure is suggested, as shown in Fig. 10, and identified as 9-ethyl-1,4,6,9,10-pentahydroxy-7,8,9,10-tetrahydrotetracene-5,12-dione with a molecular formula of C_20_ H_18_ O_7_ and a molecular weight of 370.4 g/mol.

## 4 Discussion

Large-scale biotechnological production of novel compounds with pharmaceutical significance continues to be difficult to achieve owing to the complex life cycle, multicellular characteristics, and problematic genetics of *Streptomyces* species, despite ongoing efforts in identification and characterization. Ongoing endeavors to optimize reprogramming for enhanced production and elimination of secondary metabolites have laid the foundation for the development of subsequent generations of *Streptomyces* cell factories [31]. The conventional approach to optimizing culture conditions involves the utilization of the one-factor at a time (OFAT) method. This method remains viable so long as a reduced number of variables impact the production process [32]. However, when examining multiple variables, OFAT is insufficient to characterize the collective impact of the various factors at play and requires a substantial number of trials [33]. Researchers can use the Plackett-Burman design (PBD) and response surface methodology (RSM) as Box-Bhenken design to select the important variables and determine their optimal values, respectively, thereby overcoming these limitations [[34], [35]].

From the tested nutritional and environmental variables, elicitation, yeast extract, inoculum size, and incubation time were significant factors affecting the antibacterial activity. Multiple studies have already found that these factors improve antibiotic production. For example, Luti & Yonis, [36], reported that the highest production of phenazine in the elicited culture of *Pseudomonas aeruginosa* was attained in the culture elicited with heat-killed cells of *S. cerevisiae*. According to the findings of Wadetwar et al. [37], it was observed that the incubation of actinomycetes, which were isolated from the Nagpur area, for a fermentation duration of 7 days using an inoculum size of 10%, along with the presence of yeast extract at a concentration of 0.4% and malt extract at a concentration of 1.0%, increased antibiotic production. The effects of starting pH and incubation temperature were found to be non-significant in this screening experiment since the test was conducted in circumstances closely approximating the ideal values for both parameters [38].

To determine the most effective range of antibacterial effectiveness, the influential independent variables (elicitation, yeast extract, and inoculum size) were further investigated at three different levels utilizing RSM via the implementation of the BBD [39]. RSM is a critical statistical technique that exposes interaction among the variables and screens the optimum process parameters for beneficial responses. The use of the RSM technique has been implemented to enhance the synthesis of antibacterial compounds in various species of *Streptomyces*. *Streptomyces sindenensis* [40] and *Streptomyces alboflavus* 313 [41]. After RSM optimization, *S. baarnensis* MH-133 exhibited a 1.85-fold increase in its antibacterial action against *K. pneumonia* compared to the unoptimized culture. The adequacy of the response surface model may be assessed using the coefficient of determination (R2), which quantifies the extent to which the observed response variability is accounted for by the experimental components and their interactions. A higher R2 value, approaching 1.00, demonstrates the model's effectiveness in accurately predicting the response variable [42]. In the present study, the R2 value was determined to be 0.9224, suggesting that about 92.24% of the variance in the dependent variable can be accounted for by the model when the dependent variable is assessed with *K. pneumonia*. The testing results yielded an antibacterial activity of 26 mm against *K. pneumonia*, which is in close accordance with the highest expected value of 27 mm. This result substantiates the accuracy of the model in predicting the antibacterial efficacy associated with the metabolites generated by *S. baarnensis* MH-133.

A culture batch (35 L) was carried out to separate the antibacterial metabolites by solvent extraction, which is usually utilized for the withdrawal of active metabolites from the culture filtrate of actinomycetes [43]. Numerous studies have used organic solvents of varying polarity to extract antibacterial compounds from actinomycetes [44]. Based on the findings of this investigation, it is evident that *S. baarnensis* MH-133 generated extracellular metabolites soluble in ethyl acetate that exhibited efficacy against pathogenic bacteria. However, the solvent extracts utilized in the study, namely diethyl ether and chloroform, demonstrated only a moderate inhibitory effect. In contrast, the other solvent extracts did not exhibit any antibacterial activity. Previous studies have demonstrated that ethyl acetate extraction is the primary method for obtaining the majority of antibiotic metabolites sourced from actinomycetes [45]. Furthermore, literature have noted that the strains from which the molecule is derived, the solvent used for extraction, and the specific pathogens against which the compound is tested all contribute to the variation in the antibacterial activity of the compounds [46].

An investigation using TLC plates was conducted to determine the most suitable solvent for use as the mobile phase before employing open-column chromatography to separate the antibacterial compounds from the other metabolites involved in the crude extract of *S. baarnensis* MH-133. The careful choice of an appropriate solvent is crucial in every chemical reaction, as it has a significant impact on both the chemical reactivity and the speed of the reaction [47]. Several bioactive molecules have been isolated and purified through the utilization of the paper thin-layer method. The continued prevalence of TLC can be attributed to its practicality, cost-effectiveness, and wide range of stationary phases [48]. The results confirmed that the ethyl acetate: methanol solvent system was the most suitable, as it successfully transferred all spot contents from the baseline and partitioned the sample into two separate spots. Nasr et al. [49] isolated *S. baarnensis* and performed chemical screening of the extract of this strain on TLC, which exhibited several bands on the TLC sheet. Although both studies utilized isolate of the same genus and species, the production of metabolites appears to be distinct. Several factors, such as the controlled environment in which the isolation took place, the physiological condition of the isolate, the specific characteristics of the medium and manufacturing process, and other relevant variables, may contribute to the observed outcome [50]. Furthermore, certain chemical compounds found in *Streptomyces* niches but not in culture media are believed to play a role in activating cryptic metabolites. These chemicals function as signals in sensory systems, which in turn drive regulatory cascades that are accountable for the tuning of the secondary metabolites production [51]. The fractions obtained using CC and eluted using a combination of ethyl acetate and methanol (4:6) exhibited an antibacterial property, as seen by the presence of a solitary spot on a thin-layer chromatography (TLC) plate with a retention factor (Rf) value of 0.6. Similar findings were reported by Sunil et al. [52], who used ethyl acetate as a solvent for extracting antibacterial chemicals from the culture filtrate. Additionally, it has been shown that the metabolites were separated using thin-layer chromatography (TLC) with a solvent mixture of ethyl acetate and methanol in a ratio of 6:4. Furthermore, the use of direct bioautography revealed the existence of two active compounds with retention factors (Rf) of 0.8 and 0.4.

The MIC and MBC values of the purified compound (Ka) and control antibiotic (streptomycin) were varied according to the type of the tested bacterial strain. The MIC values of Ka ranged from 37.5 to 300 μg/ml and the MBC values ranged from 75 to 300 μg/ml while the MIC values of streptomycin, which was used as a control antibiotic in this assay, ranged from 75 to 300 μg/ml, and the MBC values were ranged from 75 to 600 μg/ml. The observed MIC values of streptomycin against the tested bacteria were high; this may be due to the capability of these ESKAPE strains to tolerate different antibiotics according to their antibiotic profiles, which were mentioned previously by Moghannem et al. [10]. Previous research has indicated that the work of Chaudhary et al. [53], reported the minimum inhibitory concentration (MIC) of bioactive metabolites derived from actinomycete isolates. The MIC was observed to be 2.5 mg/ml against *Shigella dysenteriae,* vancomycin-resistant enterococci, and *Klebsiella pneumoniae.* Additionally, the MIC was found to be 1.25 mg/ml for *Bacillus cereus* and Methicillin-resistant *Staphylococcus aureus*. Furthermore, it should be noted that the minimum inhibitory concentration (MIC) of a particular agent is not a fixed value, as it may be influenced by several factors such as the characteristics of the test organism used the size of the inoculum, the composition of the culture medium, the duration of incubation, and the level of aeration [54].

The physicochemical characteristics, in addition to spectroscopic analysis including UV, IR, HNMR, and Mass spectrum of the purified active compound (Ka) produced by *S. baarnensis* MH-133 suggested that the compound Ka exhibits aromatic characteristics and is classified under the quinone or anthracycline category. Multiple anthracyclines have been identified to possess sugar residues within their molecular structures. However, analysis of the 1H NMR data of the Ka indicated a lack of glycosides. In their study, Boudjella et al. [55], isolated three reddish compounds from the Streptosporangium strain, denoted as R1, R2, and R3. Compounds R1, R2, and R3 possess anthracycline structures and include more than three aromatic rings. Analysis of the 1H NMR data of compound R2 indicated a lack of glycosides. Anthracyclines are classified as aromatic polyketides, characterized by a cyclic polyketide backbone that has a 7, 8, 9, 10-tetrahydrotetracene-5, 12-quinone structure. The variety of secondary metabolites is determined by variations in the structure of the aglycone and the various sugar residues that are attached [56]. A diverse range of anthracycline derivatives has been identified, exhibiting a wide array of biological activities. The compounds daunorubicin, doxorubicin, idarubicin, epirubicin, zorubicin, and aclacinomycin A are derived from Streptomyces sp. [57,58].

## 5 Conclusion

The genus *Streptomyces* has played a crucial role in the production of significant therapeutic drugs and other bioactive chemicals since the peak age of antibiotic exploration. The intricate life cycle of *Streptomyces* species, their multicellular organization, and their challenging genetics pose significant obstacles to the efficient large-scale biotechnological synthesis of novel medicinal compounds, despite continuous efforts in their discovery and characterization. The ongoing endeavors in this field include reprogramming techniques aimed at augmenting production and isolating secondary compounds from growth. These efforts have played a crucial role in establishing the groundwork for the development of future *Streptomyces* cell factories. The findings of this study indicate that the production of metabolites by *Streptomyces baarensis* may be enhanced by optimizing the environmental and nutritional factors. Several key factors were determined to have a notable influence on the antibacterial activity of *S. baarnensis* MH-133. These factors include elicitation, yeast extract, inoculum size, and incubation length. The Box-Behnken Design (BBD) response surface technique yielded a model of considerable significance, accounting for 92.24% of the variance observed in the antibacterial response against *K. pneumonia*. The confirmation of the inhibition zone diameter at 26 mm matched the model's accurate prediction of 27 mm. While the methodology used previously, the innovative aspect of our work is the identification of critical parameters impacting the antibacterial activity of *Streptomyces baarnensis* MH-133 through the use of the statistical approaches to develop a meaningful model and the validation of the model's accuracy in predicting antibacterial responses. Furthermore, the active metabolite KA's efficacy against ESKAPE bacteria, which are classified as “priority pathogens” by the World Health Organization, emphasizes the importance of the results.

Further investigation is advised to assess the compound's potential as a therapeutic agent or lead compound for drug development, as well as to determine its efficacy against a wider variety of bacterial pathogens. Conducting an evaluation of the identified compound's toxicity and safety profile is of utmost importance to validate the safety of the compound for potential therapeutic applications.

## Ethics approval

This study did not require ethical approval or ethical approval not applicable.

## Funding

N/A.

## Availability of data and materials

Not applicable.

## Consent to participate

Written informed consent forms were collected once all study participants received information about it.

## CRediT authorship contribution statement

**Mohamed H. Kalaba:** Conceptualization, Investigation, Writing – original draft, preparation. **Gamal M. El-Sherbiny:** Conceptualization, Writing – original draft, preparation, Supervision. **Osama M. Darwesh:** Conceptualization, Supervision. **Saad A. Moghannem:** Writing – original draft, preparation, Investigation, Supervision, All authors have read and agreed to the published version of the manuscript.

## Declaration of competing interest

The authors declare that they have no known competing financial interests or personal relationships that could have appeared to influence the work reported in this paper.
